# Characteristics of patients not clinically recognized as suffering from bacteriaemia

**DOI:** 10.1186/1757-7241-21-S2-A2

**Published:** 2013-09-09

**Authors:** Anders Johan Orland Rasmussen, Anne Grethe Mølbak, Jacob Hansen-Schwartz

**Affiliations:** 1 Emergency Department Køge Sygehus, Denmark

## Background

Criteria for Systemic Inflammatory Response Syndrome (SIRS) are recognized as operational criteria for detecting a possible bacteriaemia. Given the sensitivity and specificity of the criteria, clinical judgment is crucial in detecting the condition. The aim of the study was retrospectively to identify clinical characteristics of patients not primarily thought of as having bacteriaemia, yet harboring the condition.

## Methods

Consecutive blood cultures sampled in relation to admittance through the Emergency department from 2010 to 2012 were identified. 1615 blood cultures were identified of which 229 (14%) were positive. Group 1 were sampled on admittance where a pathogen was cultured (171 patients, ‘true positive’), group 2 on admittance where a contaminant was cultured (35 patients, ‘false positive’), group 3 were not sampled on admittance, but subsequently during hospital stay and where a pathogen was cultured (23 patients, ‘false negative’), and group 4 on admittance where culture was negative (1386 patients, ‘true negative’). At random 90 patients from group 4 were selected for analysis.

Parameters recorded: Age, gender, vital signs, white blood cell count, bacterial species, and focus. Presence of diabetes, liver disease, kidney disease, chronic obstructive lung disease and cardiac disease was registered.

## Results

Significant differences between group 1 and 3:

Temperature: Group 1: 38.3 (34.2–41.2), group 3: 37.7 (36.2–40.2)

RespiratoryrRate: Group 1: 21 (5–46), group 3: 18 (11–33)

Saturation: Group 1: 95.9% (32–100), group 3: 97.5 (range 92–100)

No significant difference among groups regarding species and focus was observed. In group 3 we identified a significantly higher proportion of patients with hepatic disease and alcohol abuse, and a tendency for a higher proportion of patients with known malignant disease.

In group 1 78% and in group 3 22% fulfilled the SIRS criteria.

## Conclusion

11% of the patients suffering from bacteriaemia in our cohort were not clinically detected in the Emergency Department. Vital parameters were within normal range underscoring the difficulty to detect these patients. The study warrants attention regarding bacteriaemia in patients suffering iver disease and possibly also patients with known malignant disease.

